# Elevated gamma-glutamyl transferase has a non-linear association with incident non-alcoholic fatty liver disease in the non-obese Chinese population: a secondary retrospective study

**DOI:** 10.1186/s12944-021-01577-8

**Published:** 2021-10-25

**Authors:** Liling Wu, Man Zhang, Haofei Hu, Qijun Wan

**Affiliations:** 1 Department of Nephrology, The First Affiliated Hospital of Shenzhen University, Shenzhen, 518000 Guangdong Province China; 2 Department of Nephrology, Shenzhen Second People’s Hospital, No.3002 Sungang Road, Futian District, Shenzhen, 518000 Guangdong Province China; 3 Department of Functional Neurology, The First Affiliated Hospital of Shenzhen University, Shenzhen, 518000 Guangdong Province China; 4 Department of Functional Neurology, Shenzhen Second People’s Hospital, No.3002 Sungang Road, Futian District, Shenzhen, 518000 Guangdong Province China

**Keywords:** Gamma-Glutamyl transferase, Incident non-alcoholic fatty liver disease, Nonlinearity, Inflection point

## Abstract

**Background:**

Effective and applicable predictors of non-alcoholic fatty liver disease (NAFLD) are needed for the non-obese Chinese population. This study was undertaken to investigate: whether serum gamma-glutamyl transferase (GGT) was associated with incident NAFLD in the non-obese Chinese population.

**Methods:**

This was a retrospective cohort study that enrolled 33,153 initially NAFLD-free individuals who underwent a health examination in Wenzhou Medical Center of Wenzhou People’s Hospital from January 2010 to December 2014.

Serum GGT levels at the time of enrollment were evaluated in 11,906 persons who follow-up. The relationship between GGT levels and incident NAFLD was analyzed using Cox regression and generalized additive models after adjusting for demographic and clinical variables. In addition, Subgroup analysis was conducted, which was explored by Cox proportional hazard models. It was stated that the data had been downloaded from the DATADRYAD website.

**Results:**

Multivariable Cox regression models were used to estimate the hazard ratio (HR) for GGT with incident NAFLD after adjusted demographic and clinical variables (HR, 1.010; 95% CI, 1.007–1.012; *P* < 0.001). The incident NAFLD in the highest quartile of GGT levels was 3.653 times as high (95% confidence interval, 2.915 to 4.579) as that the lowest quartile. A non-linear relationship was firstly detected between GGT and incidence of NAFLD, which had an inflection point of GGT was 26 U/L. The effect sizes and the confidence intervals on the left and right sides of the inflection point were 1.104 (1.089–1.120) and 1.001 (0.999–1.004), respectively. In subgroup analyses, the hazard ratio for incident NAFLD remained consistent across subgroups.

**Conclusions:**

In conclusion, the GGT level in the non-obese Chinese population was statistically significantly associated with incident NAFLD. The relationship between GGT level and incident NAFLD is non-linear. When GGT level is less than 26 U/L, GGT was strong positively with incident NAFLD.

**Supplementary Information:**

The online version contains supplementary material available at 10.1186/s12944-021-01577-8.

## Background

Non-alcoholic fatty liver disease (NAFLD) is the excessive deposition of non-alcohol fat in the liver, leading to non-alcohol steatohepatitis [1, 2]. It is a growing public health burden affecting approximately one-quarter of adults worldwide [3, 4] and is considered to be the main cause of liver-related morbidity and mortality [2, 5]. Several diseases including insulin resistance further contribute to the progression of NAFLD, including type2 diabetes and metabolic syndrome.

Gamma-glutamyltransferase (GGT), which is secreted mainly by the liver, has been regarded as a biomarker of liver disease [6]. Decreased serum GGT levels are associated with the improvement of liver histology [7]. Weight loss reduces the GGT level in patients with NAFLD [8]. In Chinese, recent studies explore the decreased GGT levels associated with the improvement of metabolic disturbances after the routine treatment of NAFLD [3]. Previous research reported that the GGT level is a marker of NAFLD in patients with metabolic syndrome [9]. Up to 80% of NAFLD patients were caused by obese [10]. However, the percentage of NAFLD in lean or non-obese patients is increasing. A better biomarker to predict the progression of NAFLD is needed to be explored. The relationship between the baseline GGT and incident NAFLD should be elucidated.

In the present research, we postulate that GGT may serve as an early predictor for the incident NAFLD in the non-obese Chinese population. To test the hypothesis, we used the previously published data for secondary data analysis. We explored the relationship between GGT levels and the risk of NAFLD in the non-obese Chinese population. In addition, a generalized additive model (GAM) is further applied to study the curve relationship between GGT level and NAFLD.

## Methods

### Study design and participants

The original data is obtained free of charge from the “DATADRYAD” database. The rationale and design have been described in detail previously [11]. This was a longitudinal study conducted from January 2010 to December 2014, which enrolled 33,153 NAFLD-free individuals who underwent a health examination in Wenzhou Medical Center of Wenzhou People’s Hospital. Individuals were excluded if they reported excess alcohol consumption (> 140 g/week for men and > 70 g/week for women) [11, 12], or had a history of viral hepatitis, autoimmune hepatitis, or other known causes of chronic liver disease; a body mass index (BMI) of ≥25 kg/m^2^; a low-density lipoprotein cholesterol (LDL-C) of > 3.12 mmol/L; were taking antihypertensive agents, anti-diabetic agents or lipid-lowing agents; and were lost to follow-up or their data were missing.

According to this study, of these 16,173 participants, 4267 were excluded according to the exclusion criteria. Individuals were excluded if the GGT value was missing or an extreme value (the extreme value is defined as the mean plus/minus three standard deviations) [13]. This study included 11,906 non-obese individuals without NAFLD. Approximately 2044 participants developed NAFLD during follow-up.

### Diagnosis of NAFLD

The ultrasound diagnostic criteria for NAFLD have been described in detail previously [14].

### Data collection

Baseline clinical examinations were performed as described previously [11]. The biochemical measurements were measured by an automated analyzer (Abbott AxSYM) using standard methods.

### Follow-up and outcome definitions

During the observation period, a follow-up evaluation will be carried out annually. The outcome was the incident NAFLD, diagnosed by ultrasonic examination.

### Statistical analysis

The purpose of multiple imputations was to generate possible values for missing values, which had been used in multivariate data sets. The participants were stratified by quartiles of GGT. Continuous variables were expressed as the means ± standard deviations or as medians and interquartile ranges, and categorical variables were expressed as proportions. Categorical variables were compared by chi-square test, and continuous variables were compared by one-way analysis of variance or Kruskal-Wallis.

The Cox proportional hazards model was used to estimate the hazard ratio (HR) and 95% confidence interval (CI) of the NAFLD risk associated with GGT levels, without and with controlling for the impact of key clinical confounding variables. A sensitivity analysis was conducted by converted the GGT into a categorical variable. A smooth curve fitting (penalty curve method) and a GAM were applied to address the relationship of GGT and the incident NAFLD. As the non-linearity relationship was observed, a two-piecewise linear regression model was performed to calculate the threshold effect of the GGT on the incident NAFLD in terms of the smoothing plot. The best-fitting model was determined based on the *P* value of the log of the likelihood ratio. A subgroup analysis was conducted, which was explored by Cox proportional hazard models with adjustment. NAFLD-free survival curves were used by the Kaplan-Meier method. The log-rank test was used to compare the Kaplan–Meier hazard ratios. The analyses were performed by package R (http://www.R-project.org, The R Foundation) and Empower-Stats (http://www.empowerstats.com, X&Y Solutions, Inc., Boston, MA). Two-tailed *P* values of less than 0.05 were considered to indicate statistical significance.

## Results

### Study participants and the baseline characteristics

**Fig. 1:**
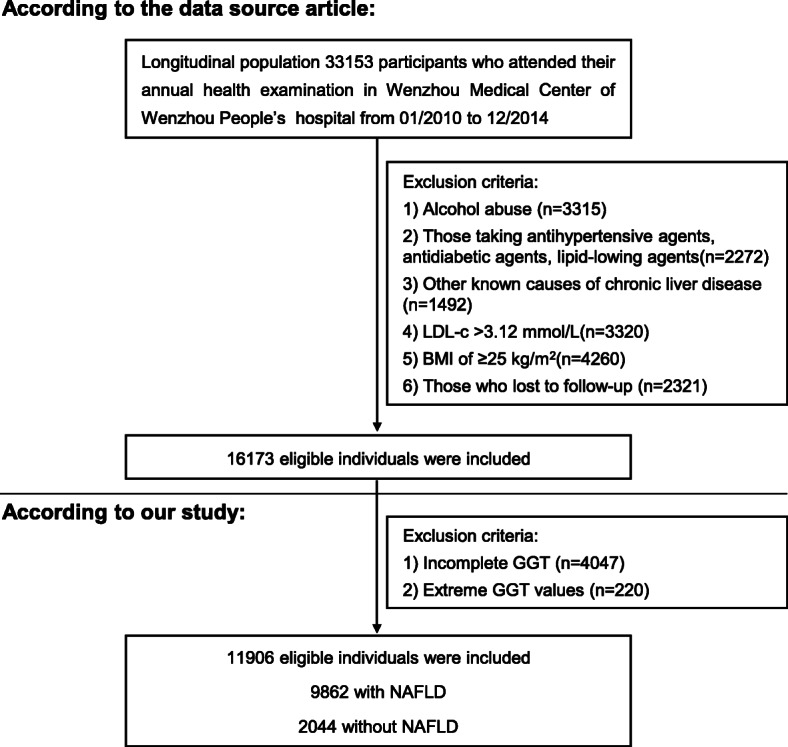
Study design and participant flow

11,906 participates (54.69% male and 45.31% female) who met the inclusion standards were enrolled (Fig.1). Participant’s age varied from 14 to 95 years old, with an average of 43.26 years old. 2044 participants developed NAFLD during follow-up. The baseline characteristics were stratified by the quartiles of the GGT (<16 U/L, 16-21 U/L, 21-31 U/L, > 31 U/L), which were shown in Table 1. Participants with the highest GGT had higher BMI, SBP, DBP, FPG, UA, TC, LDL-C, Cr, ALT and AST.

**Table 1:** The baseline characteristics of participants

GGT (U/L)	Q1 (<16)	Q2 (16 to<21)	Q3 (21 to<31)	Q4 (≥31)	***P***-value
Participants	2401	3077	3448	2980	
Age, years	42.63 ± 14.96	43.06 ± 15.01	43.39 ± 15.12	43.81 ± 14.63	0.028
Gender -n (%)					< 0.001
Female	1231 (51.27%)	1416 (46.02%)	1524 (44.20%)	1224 (41.07%)	
Male	1170 (48.73%)	1661 (53.98%)	1924 (55.80%)	1756 (58.93%)	
BMI (kg/m^2^)	20.76 ± 1.93	21.17 ± 2.01	21.84 ± 2.01	22.42 ± 1.81	< 0.001
SBP (mmHg)	115.36 ± 15.70	119.93 ± 16.52	124.32 ± 16.26	127.66 ± 16.55	< 0.001
DBP (mmHg)	69.48 ± 9.39	72.20 ± 9.75	74.92 ± 10.14	77.11 ± 10.38	< 0.001
TC (mmol/L)	4.42 ± 0.70	4.54 ± 0.72	4.62 ± 0.72	4.79 ± 0.77	< 0.001
TG (mmol/L)	0.89 (0.70–1.14)	1.01 (0.79–1.33)	1.22 (0.92–1.65)	1.52 (1.11–2.16)	< 0.001
HDL-c (mmol/L)	1.55 ± 0.35	1.49 ± 0.35	1.41 ± 0.35	1.36 ± 0.35	< 0.001
LDL-c (mmol/L)	2.13 ± 0.45	2.24 ± 0.47	2.32 ± 0.47	2.36 ± 0.46	< 0.001
ALT (U/L)	12.00 (10.00–16.00)	14.00 (11.00–18.00)	17.00 (14.00–23.00)	23.00 (17.00–32.00)	< 0.001
AST (U/L)	19.00 (17.00–22.00)	20.00 (18.00–23.00)	21.50 (19.00–25.00)	24.00 (21.00–29.00)	< 0.001
ALP (mmol/L)	62.52 ± 17.67	68.98 ± 19.42	74.28 ± 21.08	79.31 ± 25.74	< 0.001
ALB (U/L)	44.15 ± 2.75	44.49 ± 2.73	44.70 ± 2.78	44.71 ± 2.79	< 0.001
GLB (g/L)	29.01 ± 3.99	29.35 ± 3.97	29.39 ± 3.82	29.45 ± 4.07	< 0.001
TB (umol/L)	10.90 (8.30–14.10)	11.02 (8.40–14.30)	11.70 (8.91–14.90)	11.80 (9.20–15.20)	< 0.001
DBIL (umol/L)	2.00 (1.40–2.75)	2.02 (1.44–2.78)	2.10 (1.50–2.80)	2.08 (1.44–2.83)	0.211
BUN (mmol/L)	4.34 ± 1.39	4.51 ± 1.33	4.67 ± 1.33	4.77 ± 1.55	< 0.001
Cr (umol/L)	74.30 ± 18.51	80.15 ± 19.96	86.74 ± 20.85	90.12 ± 34.70	< 0.001
UA (umol//L)	240.63 ± 78.66	273.12 ± 83.65	303.91 ± 79.35	339.09 ± 84.52	< 0.001
FBG (mmol/L)	5.02 ± 0.54	5.13 ± 0.68	5.24 ± 0.83	5.39 ± 1.06	< 0.001

*ALB* albumin, *ALP* alkaline phosphatase, *ALT* alanine aminotransferase, *AST* aspartate aminotransferase, *BMI* body mass index, *BUN* blood urea nitrogen, *Cr* creatinine, *DBIL* Direct Bilirubin, *DBP* diastolic blood pressure, *FPG* fasting plasma glucose, *GGT* γ-glutamyl transpeptidase, *GLB* globulin, *HDL-c* high-density lipoprotein cholesterol, *LDL-c* low-density lipoprotein cholesterol, *SBP* systolic blood pressure, *TB* total bilirubin, *TC* total cholesterol, *TG* triglyceride, *UA* uric acid

### Univariate analyses

Perform univariate Cox proportional hazards analysis to compare the role of GGT and other variables in predicting NAFLD (Table 2). In univariate analysis, the results showed that age, BMI, TC, TG, LDL, SBP, DBP, AST, ALT, ALP, BUN, Cr, UA and FBG were positively correlated with the probability of NAFLD.

**Table 2:** The results of univariate COX regression

	Statistics	HR(95%CI)	***P***-value
Age, years	43.26 ± 14.94	1.01 (1.00, 1.01)	< 0.001
Gender -n (%)			0.2083
Female	5395 (45.31%)	Ref	
Male	6511 (54.69%)	1.06 (0.97, 1.15)	
BMI (kg/m2)	21.60 ± 2.04	1.68 (1.63, 1.73)	< 0.001
SBP (mmHg)	122.22 ± 16.88	1.01 (1.01, 1.02)	< 0.001
DBP (mmHg)	73.67 ± 10.33	1.03 (1.03, 1.04)	< 0.001
TC (mmol/L)	4.60 ± 0.74	1.28 (1.22, 1.35)	< 0.001
TG (mmol/L)	1.36 ± 0.92	1.19 (1.17, 1.20)	< 0.001
HDL-c (mmol/L)	1.45 ± 0.36	0.27 (0.23, 0.31)	< 0.001
LDL-c (mmol/L)	2.27 ± 0.47	1.78 (1.61, 1.97)	< 0.001
ALT (U/L)	19.59 ± 15.49	1.01 (1.01, 1.01)	< 0.001
AST (U/L)	22.72 ± 8.78	1.01 (1.01, 1.01)	< 0.001
ALP (mmol/L)	71.80 ± 22.14	1.01 (1.01, 1.01)	< 0.001
ALB (U/L)	44.54 ± 2.77	0.99 (0.98, 1.01)	0.4209
GLB (g/L)	29.32 ± 3.96	1.02 (1.01, 1.03)	< 0.001
TB (umol/L)	12.04 ± 5.07	0.99 (0.99, 1.00)	0.1830
DBIL (umol/L)	2.22 ± 1.17	0.68 (0.65, 0.71)	0.437
BUN (mmol/L)	4.58 ± 1.41	0.88 (0.85, 0.91)	< 0.001
Cr (umol/L)	83.37 ± 25.16	1.00 (1.00, 1.00)	< 0.001
UA (umol//L)	292.00 ± 88.80	1.00 (1.00, 1.00)	< 0.001
FBG (mmol/L)	5.21 ± 0.82	1.22 (1.19, 1.25)	< 0.001

### Relationship between GGT levels and incident NAFLD during follow-up

**Fig. 2:**
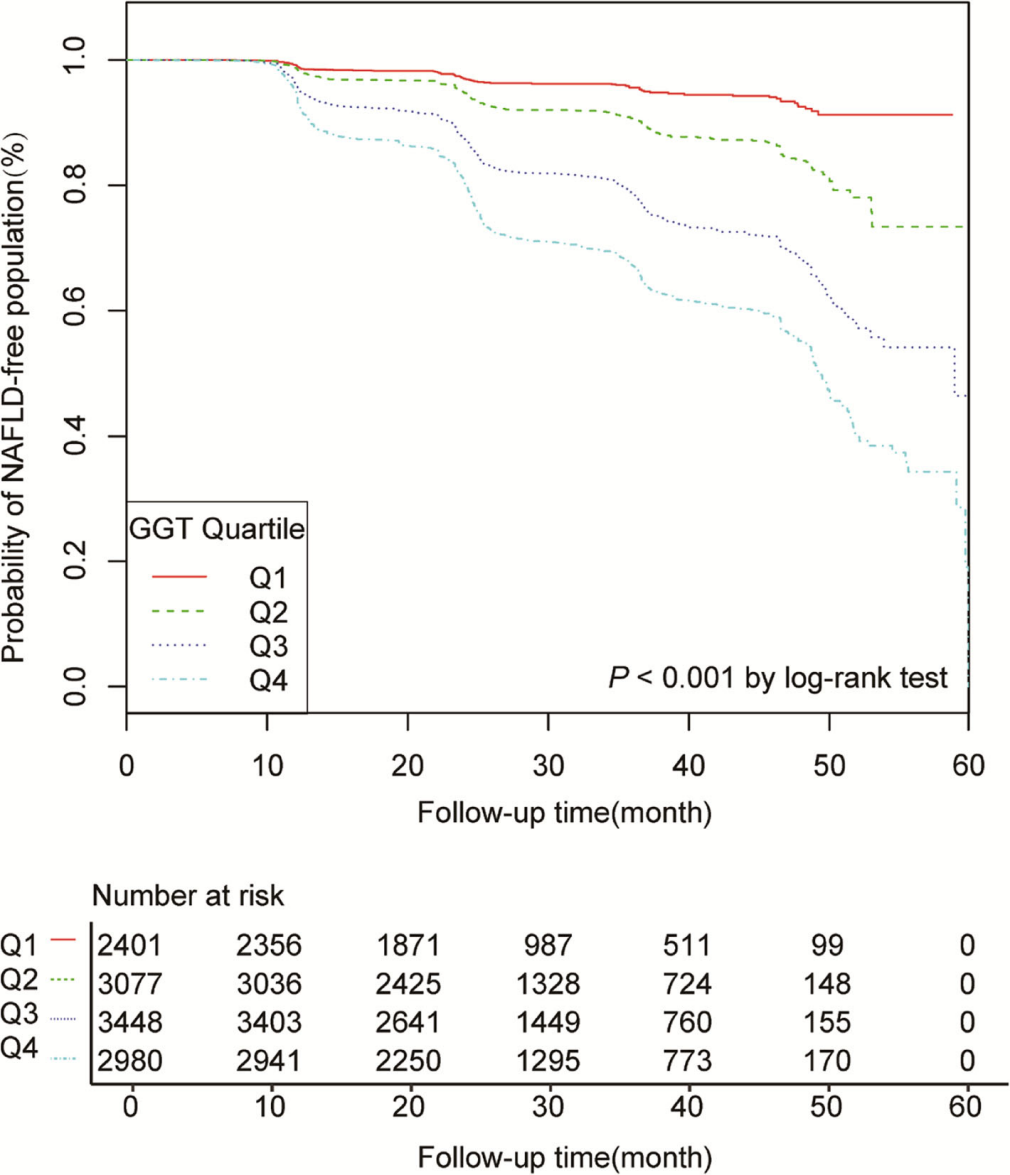
Kaplan–Meier analysis of NAFLD-free population based on GGT quartiles

Among 11,906 participants of the study cohort who had a median follow-up period of 24.97 (21.88–38.10) months. NAFLD developed in 2044 participants (17.17%) during follow-up. Higher GGT levels at baseline are associated with higher incidence of NAFLD (*P* < 0.001) (Fig.2). The rate of NAFLD was 35.22% at 2 years among participants with a GGT level of at least 21 U/L (third and fourth quartiles), compared with 8.82% among participants with a GGT level of less than 21 U/L (first and second quartiles).

Participants with a GGT level in the third quartile had a risk of incident NAFLD that was 2.88 times (HR 2.88, 95%CI 2.310, 3.609) that in the first quartile, and participants in the fourth quartile had a risk that was 3.653 times (HR 3.653, 95%CI 2.915, 4.579) that in the first quartile (Table 3). This association of GGT and NAFLD persisted despite adjustment for age, gender, BMI, SBP and DBP (HR 1.013, 95%CI 1.011, 1.015) (model I) or inclusion of the baseline mentioned above characteristics and TC, TG, HDL-C, LDL-C, ALT, AST, ALP, ALB, GLB, TB, DBIL, BUN, Cr, UA and FBG (model II) (HR 1.010, 95%CI 1.007, 1.012) (Table 3). Then use GAM to insert the continuity covariate as a curve into the equation, which is basically consistent with Model II (HR 1.004; 95% CI 1.004, 1.007), which proves the robustness of the results (Table 3).

**Table 3:** Relationship between GGT and the incident NAFLD in different models

Variable	Crude model (HR, 95%CI, ***P***)	Model I (HR, 95% CI, ***P***)	Model II (HR, 95% CI, ***P***)	GAM (HR, 95% CI, ***P***)
GGT	1.020 (1.018–1.022)<0.001	1.013 (1.011, 1.015) <0.001	1.010 (1.007, 1.012)<0.001	1.004 (1.002, 1.007) <0.001
GGT (quartile)				
Q1	Ref	Ref	Ref	Ref
Q2	2.176 (1.717, 2.757)<0.001	1.809 (1.427, 2.293)<0.001	1.661 (1.308, 2.108)<0.001	1.402 (1.100, 1.785) <0.001
Q3	5.066 (4.075, 6.298)<0.001	3.335 (2.678, 4.154)<0.001	2.888 (2.310, 3.609)<0.001	2.103 (1.671, 2.648) <0.001
Q4	8.202 (6.625, 10.156)<0.001	4.548 (3.661, 5.650)<0.001	3.653 (2.915, 4.579)<0.001	2.347 (1.853, 2.973)<0.001
*P* for trend	< 0.001	< 0.001	< 0.001	< 0.001

### The analyses of the non-liner relationship

**Fig. 3:**
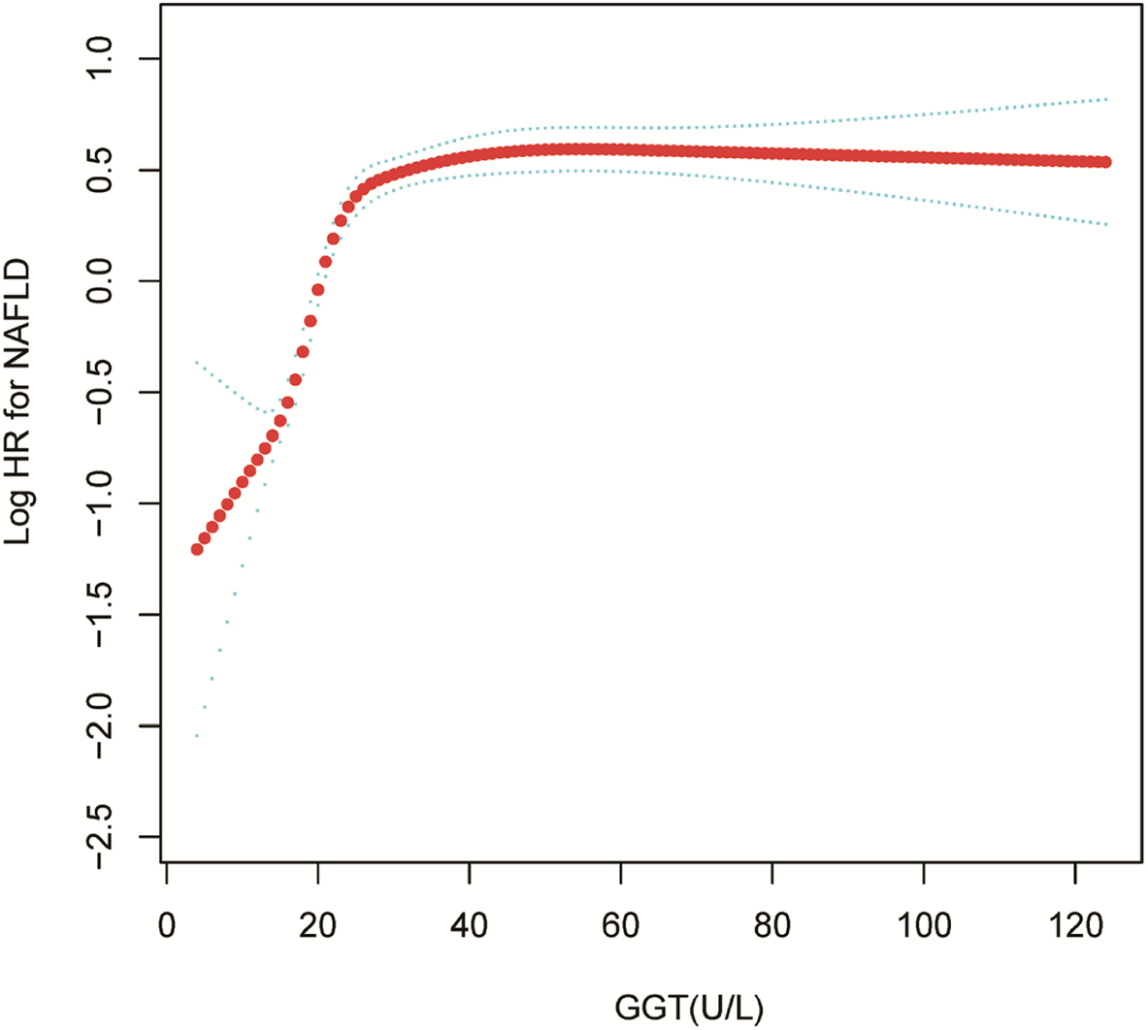
Association of GGT with the risk of NAFLD. The non-linear relationship between GGT and incident of NAFLD. A nonlinear relationship between them was detected after adjusting for age, gender, BMI, SBP, DBP, TC, TG, HDL-C, LDL-C, ALT, AST, ALP, ALB, GLB, TB, DBIL, BUN, Cr, UA, FBG

**Fig. 4:**
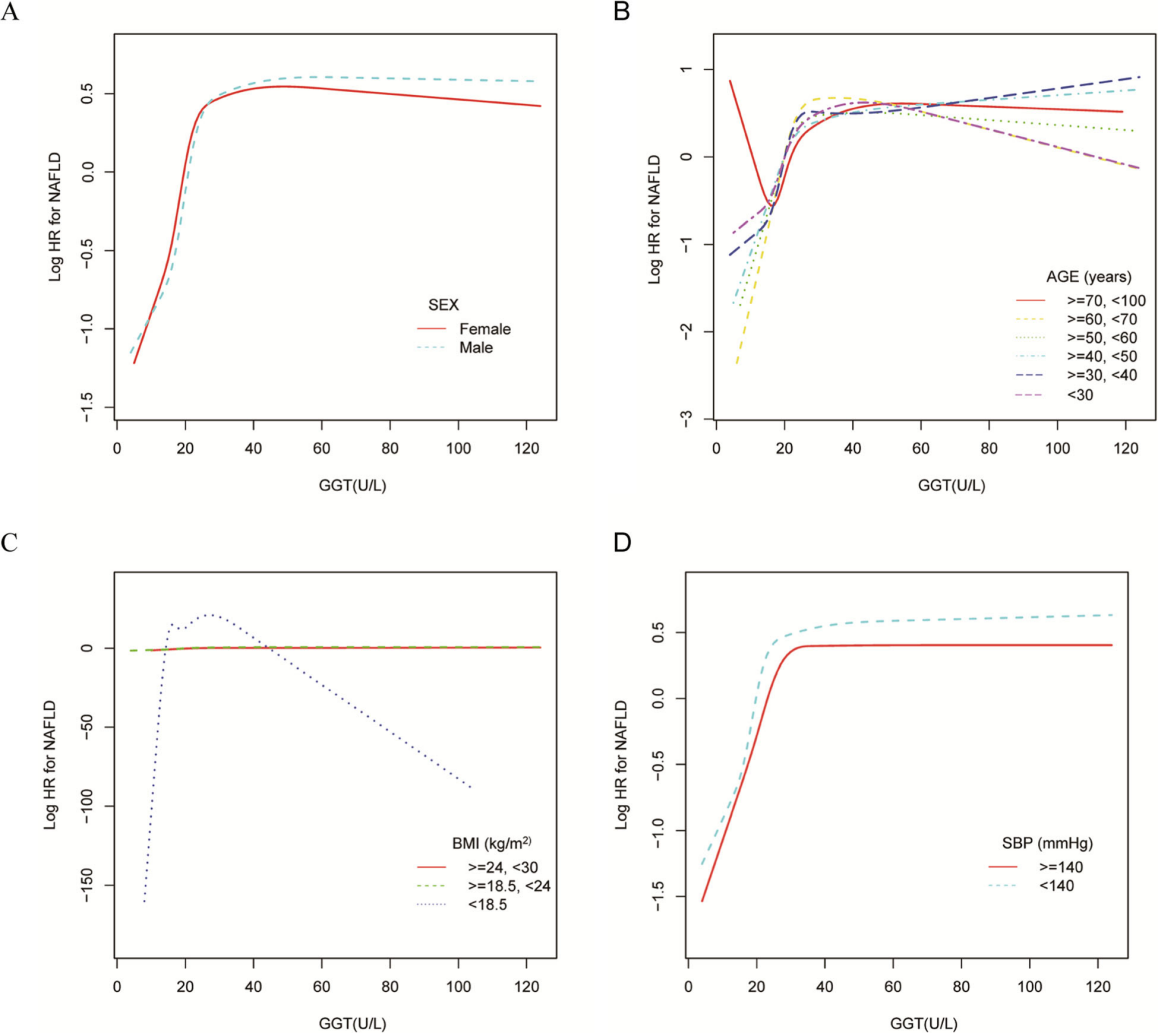
Association of GGT with the risk of NAFLD in subgroups analysis The non-linear relationship between GGT and incident of NAFLD according to subgroups in the adjusted analysis. Note 1: Above model adjusted for age, gender, BMI, SBP, DBP,, TC, TG, HDL-C, LDL-C, ALT, AST, ALP, ALB, GLB, TB, DBIL, BUN

The GGM and smooth curve fitting were applied to study the relationship between GGT level and the incidence NAFLD. Therefore, a non-linear relationship of GGT level with incidence NAFLD was detected adjusting for confounding variables, which has not been detected in previous studies. (Fig.3). Meanwhile, the curve fitting graphs were made in the subgroup analysis (Fig.4 and Supplemental Fig. 2). The two-piecewise linear regression model and recursive algorithm calculated the inflection point of GGT (Log-likelihood ratio test *P* < 0.001). When GGT levels were ≤ 26 U/L, a 1 U/L increase in GGT level is accompanied by a 10.4% increase in HR for NAFLD (HR 1.104; 95% CI 1.089, 1.120) and when GGT levels were >26 U/L, GGT have not been observed to be associated with incident NAFLD (HR 1.001; 95% CI 0.999, 1.004, *P*: 0.364) (Table 4). There was a threshold effect between GGT and incident NAFLD.

**Table 4:** The result of two-piecewise linear regression model

Incident NAFLD	HR (95% CI)	***P***
Fitting model by standard linear regression	1.010 (1.007–1.012)	<0.001
Fitting model by two-piecewise linear regression		
Inflection point of GGT		
≤26 U/L	1.104 (1.089–1.120)	<0.001
> 26 U/L	1.001 (0.999–1.004)	0.3640
*P* for log likelihood ratio test	<0.001	

We adjusted age, gender, BMI, SBP, DBP, TC, TG, HDL-C, LDL-C, ALT, AST, ALP, ALB, GLB, TB, DBIL, BUN, Cr, UA, FBG

*CI* Confidence interval, *HR* hazard ratio, *NAFLD* non-alcoholic fatty liver disease, *GGT* γ-glutamyl transpeptidase

### The results of subgroup analyses

Sensitivity analyses were also performed to determine whether age, gender, BMI, SBP, DBP, HDL-C, ALT, Cr, UA, and FBG influenced the relationship between the GGT level and the incidence of NAFLD. In subgroup analyses, the hazard ratio for incident NAFLD remained consistent across subgroups (Table 5). These findings suggest that GGT is independently associated with the incidence of NAFLD.

**Table 5:** Effect size of GGT on NAFLD in prespecified and exploratory subgroups

Characteristic	No of participants	HR (95%CI)	***P*** value	***P*** for interacion
**Age, years**				0.5082
< 30	2251	1.005 (0.999, 1.010)	0.1221	
30 to < 40	3549	1.010 (1.006, 1.014)	< 0.001	
40 to < 50	2700	1.011 (1.006, 1.016)	< 0.001	
50 to < 60	1570	1.009 (1.003, 1.015)	0.003	
60 to < 70	826	1.005 (0.996, 1.014)	0.272	
≥ 70	1010	1.010 (1.003, 1.018)	0.009	
**Gender**				0.2162
Female	5395	1.008 (1.005–1.011)	< 0.001	
Male	6511	1.011 (1.008–1.013)	< 0.001	
**BMI (kg/m**^**2**^**)**				0.1886
< 18.5	942	1.038 (0.932, 1.155)	0.499	
≥ 18.5, < 24	9377	1.013 (1.010, 1.015)	< 0.001	
≥ 24	1587	1.009 (1.005, 1.012)	< 0.001	
**SBP (mmHg)**				0.9399
< 140	10,188	1.009 (1.007–1.012)	< 0.001	
≥ 140	1718	1.010 (1.005–1.014)	< 0.001	
**DBP (mmHg)**				0.7567
< 90	10,941	1.010 (1.007–1.012)	< 0.001	
≥ 90	965	1.010 (1.005–1.016)	< 0.001	
**HDL-c (mmol/L)**				0.6828
<1	1014	1.007 (1.001, 1.013)	0.0179	
≥ 1	10,892	1.009 (1.006, 1.011)	< 0.001	
**ALT (U/L)**				0.0011
≤ 40	11,314	1.012 (1.010, 1.015)	< 0.001	
>40	592	1.003 (0.998, 1.008)	0.1828	
**Cr (mmol/L)**				0.0769
< 82	5919	1.007 (1.003, 1.010)	< 0.001	
≥ 82	5987	1.011 (1.008, 1.014)	< 0.001	
**UA (umol//L)**				0.3937
<420	10,962	1.010 (1.008, 1.012)	< 0.001	
≥ 420	944	1.007 (1.002, 1.013)	0.007	
**FBG (mmol/L)**				0.2557
<6.1	11,074	1.010 (1.008, 1.013)	< 0.001	
≥ 6.1	832	1.007 (1.002, 1.012)	0.009	

Note 1: Above model adjusted for age, gender, BMI, SBP, DBP,, TC, TG, HDL-C, LDL-C, ALT, AST, ALP, ALB, GLB, TB, DBIL, BUN, Cr, UA, FBG.

Note 2: In each case, the model is not adjusted for the stratification variable

## Discussion

This study in the non-obese Chinese population provided evidence that the baseline GGT level was associated with incident NAFLD during follow-up. Concurrently, a saturation effect was detected between the GGT level and incident NAFLD, with an inflection point at 26 U/L. Moreover, in multivariate analysis of the subgroup, GGT was also as an independent biomarker to predict the incidence of NAFLD.

NAFLD is a world public health problem [15], which is accompanied by liver dysfunction. The abnormal of liver enzymes are the markers of NAFLD in the general population [16]. As reports, the abnormal GGT is associated with the future development of the fatty liver [17]. This study showed that the baseline GGT predicted the incident NAFLD during follow in the non-obese Chinese population, which supports the previous similar studies. From a traditional perspective, central obesity and metabolic syndrome increase the risk of NAFLD [18, 19]. However, the percentage of non-obese patients with NAFLD is now growing [20]. This study first investigated the association between GGT and NAFLD in a large non-obese Chinese population. Previous studies used propensity scores to divide GGT into two categorical variables, which would greatly damage the variable’s information [9]. Moreover, previous studies did not further explore the possible curvilinear relationship between GGT and NAFLD. On the contrary, as a continuous variable, GGT (increase by 1 U/L) has a significant association with NAFLD after adjusting for confounding variables (HR = 1.010, 95% CI 1.007, 1.012). The highest quartile of GGT was associated with increased risk for NAFLD by 2.653-fold compared with the lowest quartile. Although the conclusions are consistent with previous studies, they are more ideal from the perspective of information preservation. Meanwhile, this study conducted in a large sample of the non-obese Chinese population, and the results of the study are expected to be successfully promoted among the Chinese population undergoing a physical examination. Elevated GGT reminds the population of the high risk of NAFLD during follow up, which will be an alarm for people to adjust their living habits in advance to reduce the incidence of NAFLD [21].

A GAM and smooth curve fitting were applied to study the nonlinear relation between GGT level and incident NAFLD. Two piecewise linear regressions were used to determine the relationship in detail. In the present study, the inflection point was 26 U/L after adjusting the confounding variables (including age, gender, BMI, SBP, DBP, TC, TG, HDL-C, LDL-C, ALT, AST, ALP, ALB, GLB, TB, DBIL, BUN, Cr, UA, FBG). When GGT levels were ≤ 26 U/L, a 1 U/L increase in the GGT level was associated with a 10.4% greater adjusted HR of incident NAFLD (HR 1.104; 95% CI 1.12, 5.87), and when GGT levels were >26 U/L, no relationship was observed with incident NAFLD.

In the highest baseline GGT quartile, many potentially alcohol-related variables were abnormal. The smooth curve fitting was used to show the relationship between GGT and liver examination, blood pressure and blood lipids (Supplemental Fig. 1). GGT level was positively correlated with the liver examination, blood pressure and blood lipids, the above indicators are potential alcohol-related variables, which are the highest among the highest baseline GGT quartiles. This may be related to excessive alcohol intake. However, previous research demonstrated that the GGT was strongly positively correlated to total and LDL cholesterol, triglycerides in young healthy adults [22]. The serum GGT was positively associated with uric acid, SBP, and DBP in normotensive Chinese adults. In middle-aged and elderly (females who were > 40 years old) Chinese females, the serum GGT level is associated with the UA level [23]. The above results suggest that the abnormality of these indicators (liver examination, blood pressure and blood lipids) may also be related to metabolism. The curve fitting graphs were applied in the subgroup analysis (Fig.4 and Supplemental Fig. 2). The results suggest that BMI may affect the relationship between GGT and NAFLD. However, in this study, multivariable Cox regression models were used to estimate the hazard ratio (HR) for GGT with incident NAFLD after adjusting for confounding variables (including SBP, DBP, TC, LDL, UA and drinking status), which showed GGT is an independent risk factor for NAFLD. Furthermore, the hazard ratio for incident NAFLD remained consistent across subgroups in sensitivity analysis.

### Comparisons with other studies and what does the current work add to the existing knowledge

Previous studies have shown that elevated GGT levels are the risk of NAFLD in patients with metabolic syndrome or obesity [24, 25]. However, this study was conducted in a large cohort of non-obese Chinese, allowing for subgroup analysis and adjustment of clinical confounding variables. Meantime, the non-linear relationship between NAFLD and GGT levels was investigated in different populations (with GGT above or below the inflection point). The inflection point firstly provides evidence for the management of GGT in non-obese Chinese population for the first time. Therefore, this analysis has more excellent clinical value.

### Study strength and limitations

The present study has some advantages. Firstly, GGT is used as a categorical variable and continuous variable for statistical analysis, which greatly protects the integrity of the data and enhances the robustness of the results. Secondly, we considered the possibility of the curve relationship and made a curve adjustment when adjusting confounding factors. The present study also has some limitations. Firstly, the cohort of this study included only the Chinese population, a validation study from multi-ethnic subjects was required. Secondly, the diagnosis of NAFLD was dependent on ultrasonography but not liver biopsy. Thirdly, the association between the GGT level and the different stages of NAFLD could not be done. Lastly, in this study, alcohol consumption data were obtained through questionnaires. Questionnaires have limitations, especially if patients find it embarrassing to admit that they drink too much. Since this present study is the second retrospective study, there were no data on breath, blood or urine alcohol and Big MCV. Therefore, the direct influence of excess alcohol intake on GGT levels remains unclear. At the same time, there was no data on the development of diabetes or metabolic syndrome after the follow-up period. Therefore, the influence of diabetes or metabolic syndrome on GGT levels remains unclear and needs further investigation.

## Conclusions

This present study demonstrates a positive and non-linear relationship between GGT and incident NAFLD in the non-obese Chinese population. Elevated GGT levels are associated with incident NAFLD before ultrasound diagnosis. There is a threshold effect between the GGT level and NAFLD. When GGT is lower than 26 U/L, it is positively correlated with the occurrence of NAFLD. This result is expected to provide reference for the clinicians to control GGT. From a treatment perspective, it makes sense to reduce the GGT level below the inflection point. Reducing the GGT level can significantly reduce the risk of progression to NAFLD when the GGT level is below the inflection point. Thus, abnormal GGT supports identifying the non-obese Chinese population at high risk of NAFLD, which would help clinicians plan and initiate the appropriate management strategies in advance.

## Supplementary Information

**Additional file 1.**

## Data Availability

Data can be downloaded from ‘DATADRYAD’ database (www.Datadryad.org).

## Abbreviations

ALB: Albumin
ALT: Alanine aminotransferase
ALP: Alkaline phosphatase
AST: Aspartate aminotransferase
BMI: Body mass index
BUN: Blood urea nitrogen
CIs: Confidence intervals
Cr: Creatinine
DBP: Diastolic blood pressure
DBIL: Direct bilirubin
FPG: Fasting plasma glucose
GAM: Generalized additive model
GLB: Globulin
GGT: Gamma-glutamyltransferas
HDL-C: High density liptein cholesterol
LDL-C: Low density liptein cholesterol
NAFLD: Non-alcoholic fatty liver disease
SBP: Systolic blood pressure
TB: Total bilirubin
TC: Total cholesterol
TG: Triglyceride
UA: Uric acid
