# Supplementary material for: Elevated gamma-glutamyl transferase has a non-linear association with incident non-alcoholic fatty liver disease in the non-obese Chinese population: a secondary retrospective study
Source: Lipids Health Dis. 2021 Oct 25;20:142. doi: 10.1186/s12944-021-01577-8 (PMC8543857; doi:10.1186/s12944-021-01577-8)
Supplement: Supplementary file 1 — Additional file 1. [file 12944_2021_1577_MOESM1_ESM.pdf]

Supplementary Information

Elevated gamma-glutamyl transferase has a non-linear association with incident non-alcoholic fatty liver disease in the non-obese Chinese population: a secondary retrospective study

Liling Wu[1,2†], Man Zhang[3,4†], Haofei Hu[1,2*], Qijun Wan[1,2*]

[1]Department of Nephrology, The First Affiliated Hospital of Shenzhen University, Shenzhen 518000, Guangdong Province, China.

[2]Department of Nephrology, Shenzhen Second People's Hospital, Shenzhen 518000, Guangdong Province, China.

[3]Department of Functional Neurology, The First Affiliated Hospital of Shenzhen University, Shenzhen 518000, Guangdong Province, China.

[4]Department of Functional Neurology, Shenzhen Second People's Hospital, Shenzhen 518000, Guangdong Province, China.

Full list of author information is available at the end of the article.

[*]Correspondence: wanqijun12345@126.com and huhaofei0319@126.com

[†]Liling Wu and Man Zhang contributed equally to this work.

Total number of figures: 2

FIGURE LEGEND

Supplemental Fig. 1. The relationship between GGT and blood pressure (A and B), liver examination (C and D), blood lipids (E and F), and uric acid (G).

Supplemental Fig. 2. Association of GGT with the risk of NAFLD in subgroups analysis

The non-linear relationship between GGT and incident of NAFLD according to subgroups in the adjusted analysis. Note 1: Above model adjusted for age, gender, BMI, SBP, DBP, , TC, TG, HDL-C, LDL-C, ALT, AST, ALP, ALB, GLB, TB, DBIL, BUN, Cr, UA, FBG. Note 2: In each case, the model is not adjusted for the stratification variable.

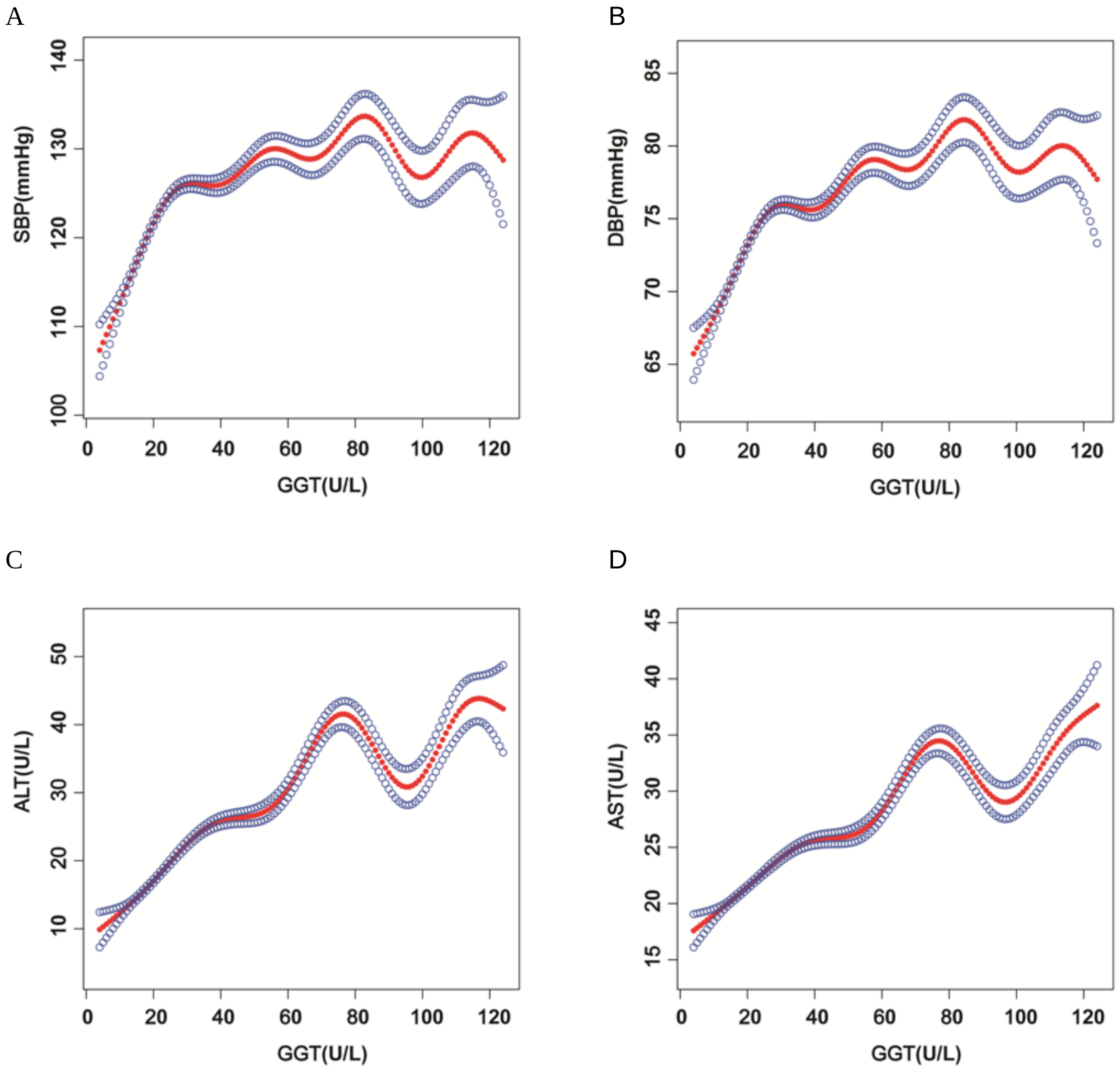
A
SBP(mmHg)
100
110
120
130
140
0
20
40
60
80
100
120
GGT(U/L)
B
DBP(mmHg)
65
70
75
80
85
0
20
40
60
80
100
120
GGT(U/L)
C
ALT(U/L)
10
20
30
40
50
0
20
40
60
80
100
120
GGT(U/L)
D
AST(U/L)
15
20
25
30
35
40
45
0
20
40
60
80
100
120
GGT(U/L)

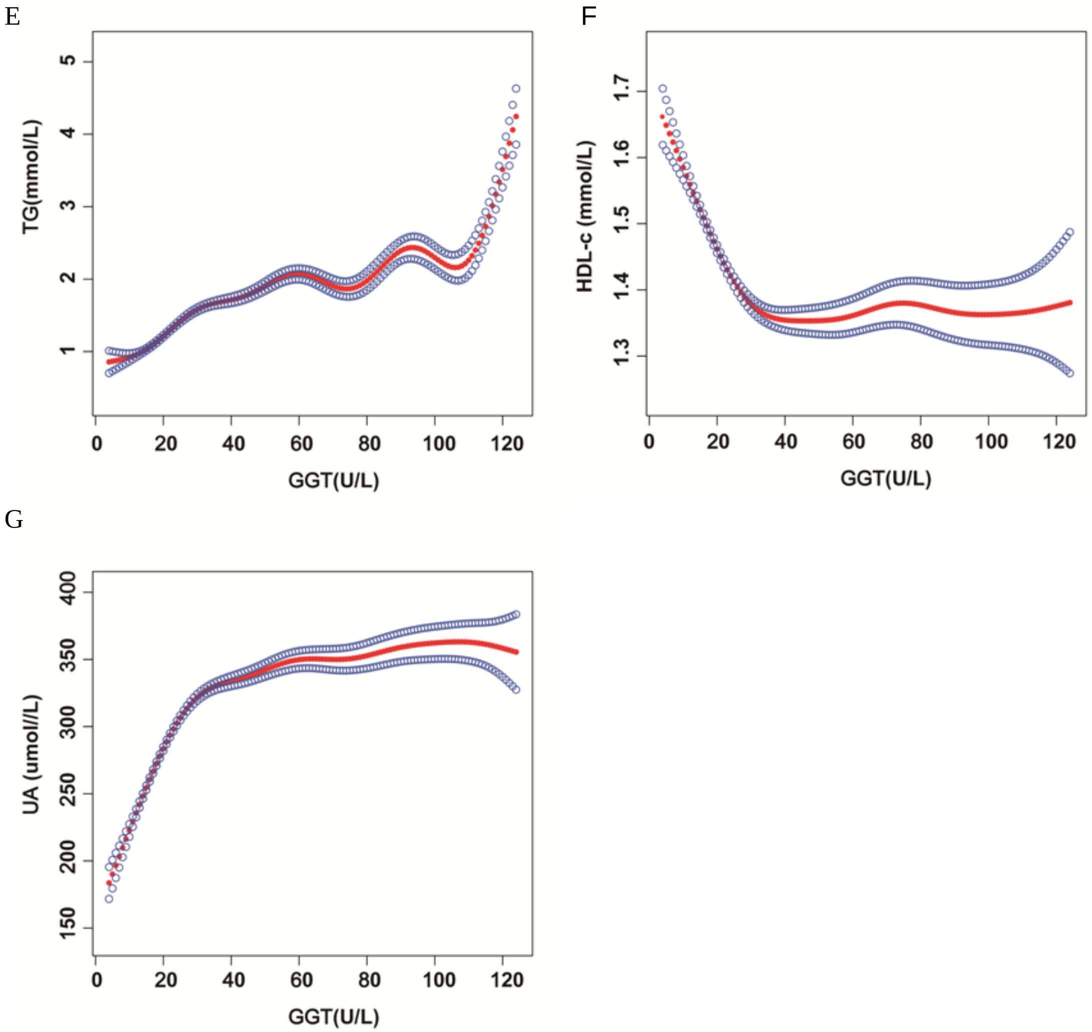

Supplemental Fig. 1. The relationship between GGT and blood pressure (A and B), liver examination (C and D), blood lipids (E and F), and uric acid (G).

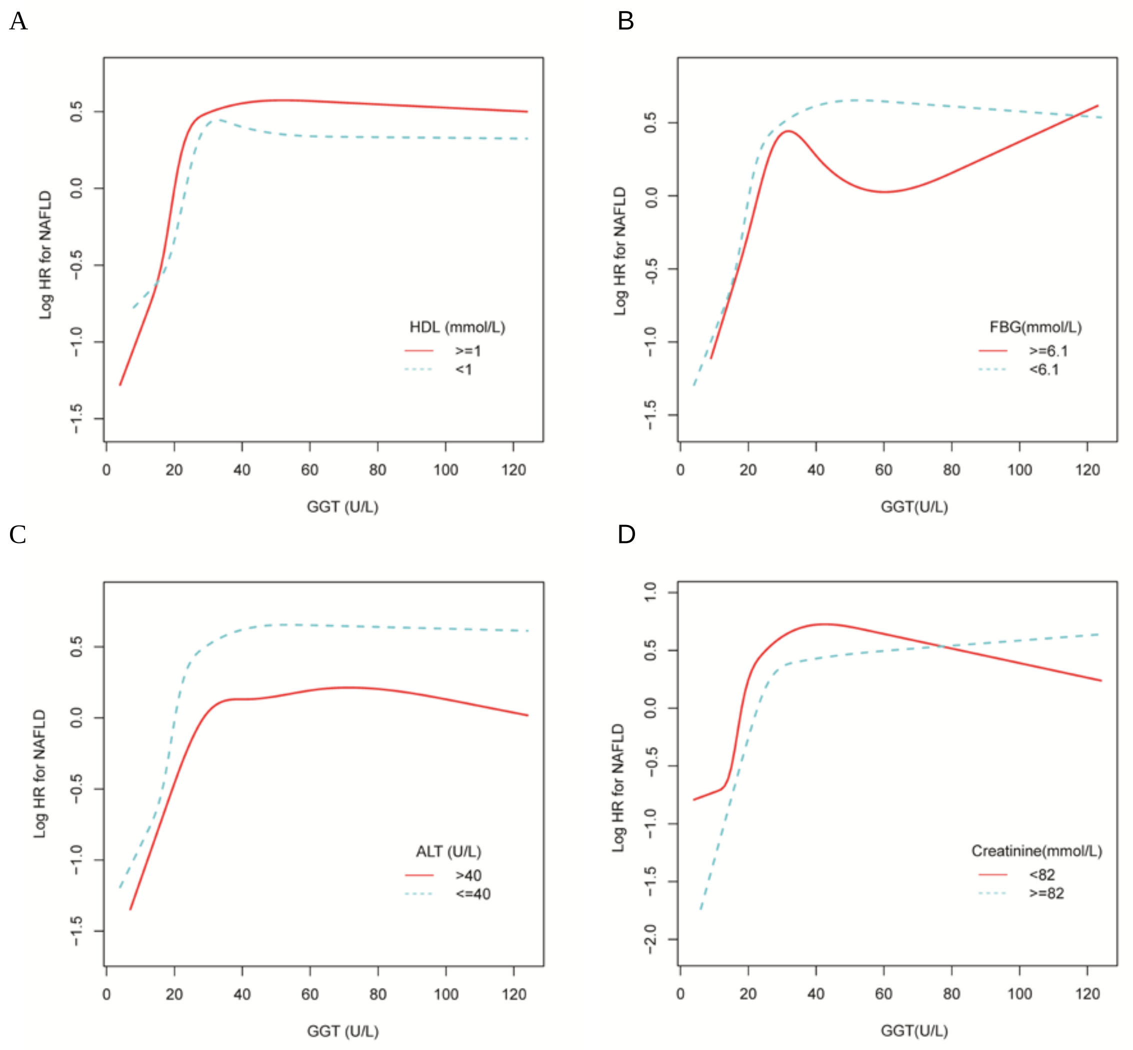

Supplemental Fig. 2. Association of GGT with the risk of NAFLD in subgroups analysis

The non-linear relationship between GGT and incident of NAFLD according to subgroups in the adjusted analysis. Note 1: Above model adjusted for age, gender, BMI, SBP, DBP, , TC, TG, HDL-C, LDL-C, ALT, AST, ALP, ALB, GLB, TB, DBIL, BUN, Cr, UA, FBG. Note 2: In each case, the model is not adjusted for the stratification variable.
